# Chronically Established ρ^0^ Cancer Cells Exhibit Shared Stemness-Associated and Aggressive Phenotypes Across Multiple Cancer Cell Lines

**DOI:** 10.7150/jca.138124

**Published:** 2026-09-03

**Authors:** Yu-Seon Han, Myeong-Eun Jegal, Su Yeon Lee, Joung-Sun Park, Yung-Jin Kim

**Affiliations:** 1 Institute of Nanobio Convergence, Pusan National University, Busan 46241, Republic of Korea.; 2 Department of Molecular Biology, Pusan National University, Busan 46241, Republic of Korea.; 3 Marine Biotechnology & Bioresource Research Department, Korea Institute of Ocean Science & Technology, Busan, Republic of Korea.

**Keywords:** mtDNA depletion, ρ^0^ cells, cancer cell plasticity, stemness-associated phenotype, cytoskeletal remodeling

## Abstract

Chronically established mitochondrial DNA-depleted (ρ^0^) cancer cells may exhibit phenotypic changes beyond metabolic dysfunction; however, whether such changes are shared across different cancer types remains unclear. In this study, we generated chronically established ρ^0^ models from pancreatic, lung, and breast cancer cell lines and examined their common phenotypic features. Compared with their parental wild-type counterparts, the ρ^0^ cell lines exhibited increased proliferation, chemoresistance-related features, sphere-forming ability, wound closure, invasion-related phenotypes, and angiogenic activity, together with common morphological alterations. These changes were accompanied by VDAC1 upregulation and cytoskeletal remodeling. Pharmacologic treatment with VBIT-4 attenuated several of these phenotypes, whereas its effects on chemoresistance-related responses varied across cell lines. Collectively, these findings suggest that chronically established ρ^0^ cancer cells exhibit shared stemness-associated and aggressive phenotypes across multiple cancer cell lines, accompanied by VDAC1 upregulation and cytoskeletal remodeling.

## Introduction

Mitochondria play essential roles in cellular energy production, biosynthesis, apoptosis, redox regulation, and intracellular signaling 1, 2. Mitochondrial dysfunction has been implicated in a wide range of diseases and is increasingly recognized as an important feature of tumor progression and cellular adaptation in cancer 2-7. However, the extent to which chronic mitochondrial DNA (mtDNA) depletion is associated with shared phenotypic changes across different cancer types remains incompletely understood.

We previously demonstrated that mtDNA depletion-associated mitochondrial dysfunction in Hep3B cells was associated with stemness-associated and aggressive cancer cell phenotypes 8. Mitochondrial pathways have also been implicated in cancer cell plasticity and stemness-associated phenotypes 9. Wnt signaling is likewise widely associated with stemness-associated phenotypes and cancer cell plasticity 10-12. Cancer cells exhibiting stemness-associated phenotypes can display properties such as sphere-forming ability, chemoresistance, wound closure and invasion-related behavior, and pro-angiogenic activity, all of which are often associated with tumor progression, recurrence, and metastasis 10-12. Whether these phenotypic changes are shared among chronically established mtDNA-depleted cancer cells derived from different tissue origins remains unclear.

Voltage-dependent anion channels (VDACs) are mitochondrial porins located on the outer mitochondrial membrane that participate in metabolite and ion transport, reactive oxygen species release, apoptotic signaling, and regulation of mitochondrial membrane permeability 13-16. The cytoskeleton, comprising microfilaments, microtubules, and intermediate filaments, maintains cell shape and structural integrity and is closely associated with mitochondrial dynamics and organelle organization 16, 17. These functional relationships suggest that alterations in mitochondrial membrane-associated factors and cytoskeletal organization may be relevant to the phenotypic changes observed in chronically established ρ^0^ cells.

In this study, we examined whether chronically established ρ^0^ cells derived from pancreatic, lung, and breast cancer cell lines exhibit shared stemness-associated and aggressive phenotypes. We further investigated whether these changes are accompanied by VDAC1 upregulation and cytoskeletal remodeling and whether pharmacologic treatment with VBIT-4 alters selected phenotypes observed in ρ^0^ cells.

## Materials and Methods

### Materials

Dulbecco's modified Eagle's medium (DMEM), RPMI 1640, and fetal bovine serum (FBS) were purchased from JBI (Daegu, Republic of Korea). EGM^®^-2 BulletKit^®^, EGM^®^ BulletKit^®^, and ReagentPack™ subculture reagents were obtained from Lonza (Basel, Switzerland). TRIzol reagent was purchased from QIAGEN (Venlo, Netherlands). Reverse Transcriptase Premix and PCR Premix were purchased from Elpis Biotech and Bioneer (Daejeon, Republic of Korea), respectively. Pro-PREP™ protein extraction solution was obtained from iNtRON Biotechnology (Daejeon, Republic of Korea). Primary antibodies were purchased from Cell Signaling Technology (Danvers, MA, USA), Proteintech (Rosemont, IL, USA), and Santa Cruz Biotechnology (Santa Cruz, CA, USA). Alexa Fluor™ 488, DAPI, MitoTracker™ Red CMXRos, and phalloidin were purchased from Invitrogen (Carlsbad, CA, USA). Doxorubicin (DX), ethidium bromide, rotenone, and antimycin A were purchased from Sigma-Aldrich (St. Louis, MO, USA). VBIT-4 was purchased from Aobious (Gloucester, MA, USA). The In Situ Cell Death Detection Kit was purchased from Roche (Indianapolis, IN, USA). Matrigel and Transwell inserts with 8-μm pores were purchased from Corning (Corning, NY, USA).

### Cell culture

Hep3B and MDA-MB-231 cells were obtained from the Korean Cell Line Bank (Seoul, Republic of Korea), and HPAC and A549 cells were obtained from the American Type Culture Collection (Manassas, VA, USA). HPAC, A549, and MDA-MB-231 cells were maintained in RPMI 1640 supplemented with 10% FBS and 25 μg/mL amphotericin B at 37℃ in a humidified atmosphere containing 5% CO_2_. Hep3B cells were maintained in DMEM supplemented with 10% FBS and 25 μg/mL amphotericin B under the same conditions. Hep3B/ρ^0^ cells had been established previously and were maintained in our laboratory, whereas HPAC/ρ^0^, A549/ρ^0^, and MDA-MB-231/ρ^0^ cells were newly established in this study. All ρ^0^ cells were maintained in DMEM supplemented with 10% FBS, 50 μg/mL uridine, 1 mM sodium pyruvate, and 25 μg/mL amphotericin B at 37℃ in 5% CO_2_.

### Human umbilical vein endothelial cell (HUVEC) culture

HUVECs (Lonza) were maintained in EGM™-2 medium supplemented with the EGM™-2 Endothelial SingleQuots™ kit at 37℃ in 5% CO_2_. Cells at passages 4-7 were used for experiments.

### Establishment of ρ^0^ cell lines

HPAC/ρ^0^, A549/ρ^0^, and MDA-MB-231/ρ^0^ cells were established by long-term treatment with ethidium bromide. Parental cells were cultured for 2-3 weeks in complete DMEM containing 1 μg/mL ethidium bromide, after which candidate ρ^0^ cells were selected by treatment with rotenone and antimycin A. After selection and confirmation of mtDNA depletion, the cells were maintained in ethidium bromide-free ρ^0^ maintenance medium. Loss of mitochondrial gene expression was used as an indicator of mtDNA depletion.

### Reverse transcriptase-polymerase chain reaction (RT-PCR)

Total RNA was isolated using TRIzol reagent according to the manufacturer's instructions. Complementary DNA was synthesized from 2 μg of total RNA using Reverse Transcriptase Premix. PCR amplification was performed in a total volume of 20 μL containing PCR Premix and gene-specific primers. The amplification conditions were as follows: 30 cycles of denaturation at 95℃ for 20 s, annealing at 57℃ for 10 s, and extension at 72℃ for 40 s. PCR products were separated by electrophoresis on 1% agarose gels, stained with GelRed™, and visualized under UV illumination. The expression of mtDNA-encoded genes (COX1, COX2, COX3, ND1, ND2, CYTB, and ATP6) was examined to assess depletion of mitochondrial transcripts in ρ^0^ cells. Primer sequences are provided in Table 1.

### Crystal violet assay

For proliferation assays, cells were seeded at 2 × 10^4^ cells/well in 24-well plates and incubated for 48 h in their respective maintenance media. For drug response assays, cells were incubated overnight and subsequently treated with 2 μM DX and/or 10 μM VBIT-4 for 48 h. Following treatment, the cells were stained with 1 mg/mL crystal violet for 10 min, washed, and dissolved in 10% SDS. Absorbance was measured at 570 nm.

### TUNEL assay

Apoptotic cell death was assessed using the In Situ Cell Death Detection Kit (Roche). Cells were incubated overnight and subsequently treated with 2 μM doxorubicin for 48 h. The cells were fixed with paraformaldehyde, permeabilized, and incubated with the TUNEL reaction mixture for 1 h at 37 °C in the dark. Fluorescence images were acquired using an iRiS™ Digital Cell Imaging System (Logos Biosystems, Gyeonggi-do, Republic of Korea) at 200× magnification.

### Western blotting

Total cell lysates were prepared using Pro-PREP™ extraction buffer supplemented with 1 mM sodium orthovanadate. Equal amounts of protein (30 μg) were separated by electrophoresis on 10% SDS-polyacrylamide gels and transferred onto polyvinylidene difluoride membranes. Membranes were incubated with appropriate primary antibodies diluted 1:1000, followed by incubation with horseradish peroxidase-conjugated secondary antibodies. Protein signals were detected using enhanced chemiluminescence reagents.

### Wound-healing assay

Cells (1 × 10^6^) were seeded in 60-mm dishes and incubated overnight. A linear wound was generated using a razor blade, after which cells were maintained for up to 24 h in medium containing 1% FBS with or without 10 μM VBIT-4. RPMI 1640 was used for HPAC, A549, and MDA-MB-231 cells, whereas DMEM was used for Hep3B and all ρ^0^ cells. Wound closure was assessed by acquiring images of wounded areas at 40× magnification between 16 and 24 h after wounding, and cells that migrated beyond the reference line were counted.

### Transwell invasion assay

Cell invasion was assessed using 24-well Transwell chambers fitted with 8-μm-pore membranes coated with Matrigel (1 mg/mL). Cells (2 × 10^4^) were suspended in medium containing 1% FBS with or without 10 μM VBIT-4 and seeded into the upper chambers. The lower chambers contained the corresponding maintenance medium for each cell line: RPMI 1640 for HPAC, A549, and MDA-MB-231 cells, and DMEM for Hep3B and all ρ^0^ cells. After 18 h of incubation, cells that had invaded to the lower surface of the membrane were fixed, stained, and counted under a microscope at 100× magnification.

### Conditioned medium (CM) preparation

Cells (1 × 10^6^) were seeded in 100-mm dishes in their respective maintenance media and incubated overnight. After washing with PBS, the medium was replaced with medium containing 1% FBS: RPMI 1640 for HPAC, A549, and MDA-MB-231 cells, and DMEM for Hep3B and all ρ^0^ cells. After 24 h, the CM was collected, centrifuged to remove debris, and filtered through a 0.22-μm filter. CM was freshly prepared for experiments or stored at -80 °C until use.

### Tube formation assay

A 96-well plate was coated with Matrigel at 60 μL/well and allowed to polymerize. HUVECs (2 × 10^4^ cells/well) were seeded onto the polymerized Matrigel layer and incubated in a mixture of EGM and CM at a 7:3 ratio. CM derived from wild-type (WT) or ρ^0^ cells was used as indicated, and 10 μM VBIT-4 was added where applicable. After 6 h of incubation, tube-like structures were imaged at 100× magnification. Quantitative analysis was performed using the Angiogenesis Analyzer plugin in ImageJ.

### Immunofluorescence staining

Cells (2 × 10^4^) were cultured on glass coverslips for 24 h with or without 10 μM VBIT-4. For mitochondrial staining, the cells were incubated with 200 nM MitoTracker for 30 min. The cells were then fixed with 3.7% formaldehyde and permeabilized with 0.1% Triton X-100. The cells were incubated with primary antibodies against VDAC1, β-tubulin, or vimentin, followed by incubation with Alexa Fluor™ 488-conjugated secondary antibodies. F-actin was visualized using phalloidin, and nuclei were counterstained with DAPI. Images were acquired using an iRiS™ Digital Cell Imaging System (Logos Biosystems) at 200× magnification.

### Sphere formation assay

Sphere-forming ability was assessed using non-adherent 96-well plates precoated with 0.4% agarose. One cell was seeded per well to minimize aggregation-derived sphere formation and cultured for 14 days in their respective maintenance media. Sphere formation was assessed based on the presence or absence of sphere formation in each well at the end of the culture period. For VBIT-4 treatment experiments, cells were exposed to 10 μM VBIT-4 throughout the culture period.

### Protein-protein interaction network analysis

Protein-protein interaction network analysis was performed using the STRING database. Selected mitochondrial, cytoskeletal, membrane-associated, and stemness-related factors were used as input based on the phenotypic and molecular changes observed in ρ^0^ cells. The resulting network was organized to better facilitate visualization of the relationships involving VDAC family proteins.

### Statistical analysis

Data are presented as mean ± standard deviation (SD). Statistical analysis was performed using GraphPad Prism software (version 9; GraphPad, San Diego, CA, USA). Two-group comparisons were performed using Student's *t*-test, whereas multiple-group comparisons were performed using one-way analysis of variance (ANOVA) followed by Tukey's multiple comparison test. Analyses involving two independent variables were performed using two-way ANOVA. Statistical significance was set at *P* < 0.05.

## Results

### Chronically established ρ^0^ cells exhibit common structural and growth-related changes

**Figure 1:**
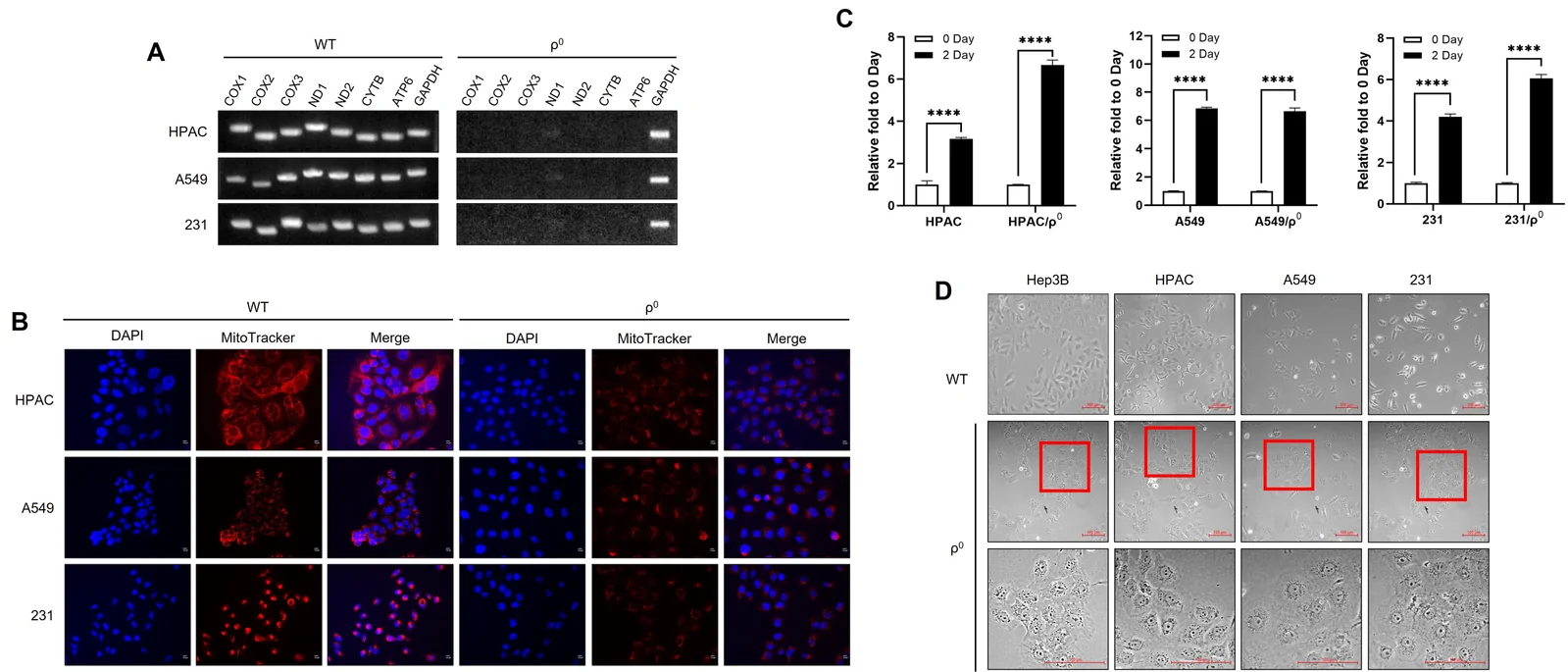
** Establishment and common features of chronically established ρ^0^ cells. (A)** RT-PCR was performed to examine mitochondrial gene expression in wild-type (WT) and ρ^0^ cells, demonstrating loss of mitochondrial gene expression in ρ^0^ cells. **(B)** MitoTracker staining was performed to compare mitochondrial content between WT and ρ^0^ cells and revealed a marked reduction in ρ^0^ cells. **(C)** Cell proliferation was assessed by crystal violet assay, demonstrating increased growth of ρ^0^ cells compared with WT cells. **(D)** Bright-field images show morphological differences between WT and ρ^0^ cells. The red boxes highlight the regions shown at higher magnification in the panels below. Data are presented as mean ± SD. ****P < 0.0001; ***P < 0.001; **P < 0.01; *P < 0.05; ns, not significant. 231 denotes MDA-MB-231.

To investigate whether chronically established mtDNA-depleted (ρ^0^) cells exhibit common phenotypic changes across different cancer types, we generated ρ^0^ cell lines from pancreatic, lung, and breast cancer cell lines. Because mtDNA depletion is a defining characteristic of ρ^0^ cells, the expression of mtDNA-encoded genes, namely *COX1, COX2, COX3, ND1, ND2, CYTB,* and *ATP6*, was examined. All established ρ^0^ cell lines exhibited complete loss of expression of these mitochondrial genes (Figure 1A). In addition, MitoTracker staining revealed markedly reduced mitochondrial content in ρ^0^ cells compared with their parental WT counterparts (Figure 1B), confirming successful establishment of the mtDNA-depleted ρ^0^ models.

Despite their different tissue origins, the ρ^0^ cell lines exhibited several common features. All ρ^0^ cell lines showed increased proliferation rates compared with WT cells, with increases of approximately 1.3- to 2-fold depending on the cell line (Figure 1C). The greatest increase was observed in the HPAC-derived ρ^0^ cells. Bright-field microscopy further revealed that WT cells exhibited distinct morphologies depending on their tissue origins, whereas ρ^0^ cells consistently exhibited a rounded and flattened morphology (Figure 1D). Collectively, these findings demonstrate that chronically established ρ^0^ cells exhibit common structural and growth-related changes across multiple cancer cell lines of different tissue origins.

### Chronically established ρ^0^ cells exhibit stemness-associated and aggressive phenotypes

**Figure 2:**
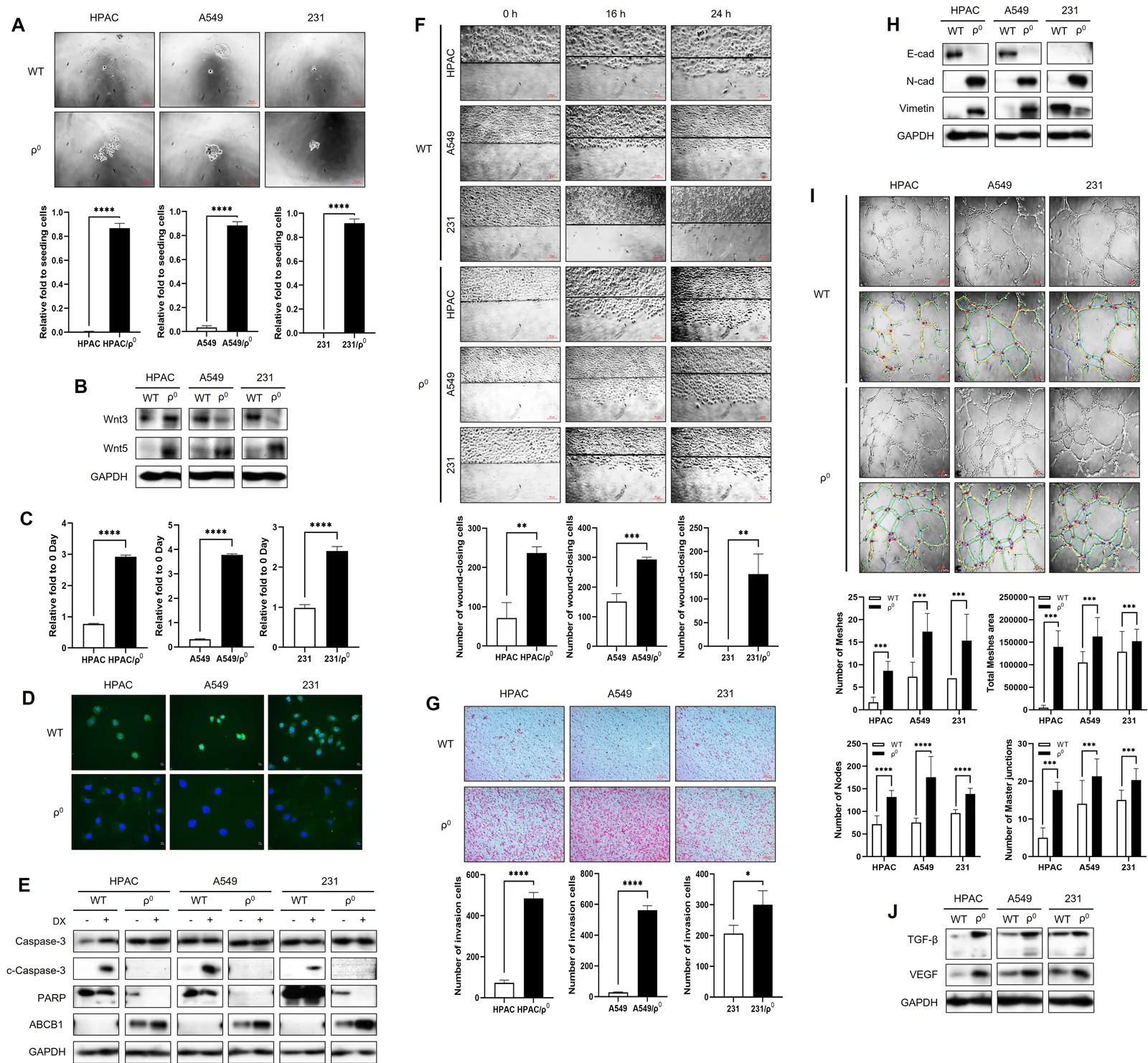
** Stemness-associated and aggressive features in chronically established ρ^0^ cells. (A)** Sphere formation was assessed under single-cell-per-well culture conditions and was observed in ρ^0^ cells but not in WT cells. The number of sphere-forming wells was quantified as a measure of sphere-forming ability. **(B)** Wnt3 expression was decreased and Wnt5 expression was increased in most ρ^0^ cell lines, whereas HPAC/ρ^0^ cells exhibited increased Wnt3 expression compared with WT cells. **(C)** Chemoresistance was assessed after treatment with doxorubicin (DX), and ρ^0^ cells exhibited greater resistance than WT cells. **(D)** TUNEL staining was performed to detect apoptotic cells following DX treatment. Apoptotic signals were observed in WT cells but not in ρ^0^ cells. **(E)** Following DX treatment, cleaved caspase-3 was detected in WT cells but not in ρ^0^ cells. The PARP antibody used recognizes both full-length and cleaved PARP; under these conditions, PARP cleavage was detected in WT cells, whereas no detectable cleaved PARP band was observed in ρ^0^ cells. ABCB1 expression was increased in ρ^0^ cells. **(F)** Wound closure was assessed using a wound-healing assay and quantified, demonstrating enhanced wound closure in ρ^0^ cells under reduced-serum conditions. **(G)** Cell invasion was assessed using a Transwell assay, and the number of invading cells was quantified, demonstrating the increased invasive ability of ρ^0^ cells. **(H)** In most ρ^0^ cell lines, E-cadherin expression was decreased, whereas N-cadherin and vimentin expression were increased; however, vimentin expression was decreased in MDA-MB-231/ρ^0^ cells. **(I)** Tube formation was assessed in human umbilical vein endothelial cells (HUVECs) exposed to WT- and ρ^0^-derived conditioned media. Quantitative ImageJ-based analysis demonstrated increased angiogenic activity in the ρ^0^ conditioned media group. **(J)** Expression of the angiogenic factors TGF-β and VEGF was increased in ρ^0^ cells. Data are presented as mean ± SD. ****P < 0.0001; ***P < 0.001; **P < 0.01; *P < 0.05; ns, not significant. 231 denotes MDA-MB-231.

We next examined whether chronically established ρ^0^ cells exhibit enhanced sphere-forming ability under single-cell culture conditions designed to minimize aggregation-derived sphere formation. None of the WT cell lines formed spheres, whereas all ρ^0^ cell lines formed spheres from single cells (Figure 2A). Although sphere morphology and size varied among the ρ^0^ cell lines, sphere-forming efficiency was consistently higher in ρ^0^ cells than in WT cells. To further characterize molecular changes associated with the ρ^0^ state, we analyzed the expression of Wnt3 and Wnt5. Most ρ^0^ cell lines exhibited decreased Wnt3 expression and increased Wnt5 expression compared with WT cells; however, HPAC/ρ^0^ cells exhibited increased Wnt3 expression compared with HPAC WT cells (Figure 2B). These findings indicate that alterations in Wnt-related signaling components associated with the ρ^0^ state vary among cell lines.

We next determined whether chronically established ρ^0^ cells exhibit altered responses to doxorubicin treatment, including chemoresistance-related features. Following doxorubicin treatment, WT cells exhibited a marked reduction in cell number, whereas ρ^0^ cells primarily exhibited reduced cell growth rather than overt cell death (Figure 2C). Consistent with this, TUNEL assays revealed TUNEL-positive cells in WT cells but not in ρ^0^ cells following doxorubicin treatment (Figure 2D). Western blot analysis further supported these findings, as cleaved caspase-3 was detected in WT cells but not in ρ^0^ cells. The PARP antibody used in this study recognizes both full-length and cleaved PARP; under our experimental conditions, PARP cleavage was detected in WT cells following doxorubicin treatment, whereas no detectable cleaved PARP band was observed in ρ^0^ cells. In addition, ABCB1 expression was higher in ρ^0^ cells under basal conditions and further increased following doxorubicin treatment (Figure 2E). Collectively, these results indicate that the chronically established ρ^0^ state is associated with enhanced chemoresistance-related features and reduced apoptotic responses to doxorubicin.

We next examined whether wound closure and invasion-related phenotypes were altered in ρ^0^ cells. Wound-healing assays showed that all ρ^0^ cell lines exhibited greater wound closure compared with WT cells under reduced-serum (1% FBS) conditions (Figure 2F), with approximately five-fold and six-fold increases observed in HPAC and A549 cells, respectively, whereas MDA-MB-231 WT cells showed little detectable wound closure under the same conditions in contrast to the marked wound closure observed in the corresponding ρ^0^ cells. Similarly, Transwell invasion assays demonstrated greater invasive activity in ρ^0^ cells, with the number of invading cells increasing by approximately 1.4- to 20-fold compared with WT cells (Figure 2G). These phenotypic changes were accompanied by molecular features consistent with a more invasive state, including decreased E-cadherin expression and increased N-cadherin and vimentin expression in most ρ^0^ cell lines (Figure 2H). Notably, E-cadherin expression was minimal in both WT and ρ^0^ MDA-MB-231 cells, and vimentin expression was reduced in MDA-MB-231/ρ^0^ cells. Collectively, these findings support the association of the chronically established ρ^0^ state with a more aggressive phenotype.

We further examined whether the ρ^0^ state affects angiogenic activity. Conditioned media from ρ^0^ cells induced greater tube formation in HUVECs than conditioned media from WT cells, as shown by representative images and quantitative analysis using ImageJ (Figure 2I). Quantitative analysis revealed significant increases in the number of nodes, master junctions, and meshes, as well as in total mesh area, in HUVECs treated with ρ^0^ conditioned media. Consistent with these findings, the angiogenic factors TGF-β and VEGF were upregulated in ρ^0^ cells (Figure 2J). Collectively, these results indicate that chronically established ρ^0^ cells exhibit multiple stemness-associated and aggressive features, including enhanced sphere-forming ability, chemoresistance-related features, wound closure, invasion, and pro-angiogenic activity.

### VDAC1 upregulation and cytoskeletal remodeling are common features of chronically established ρ^0^ cells

**Figure 3:**
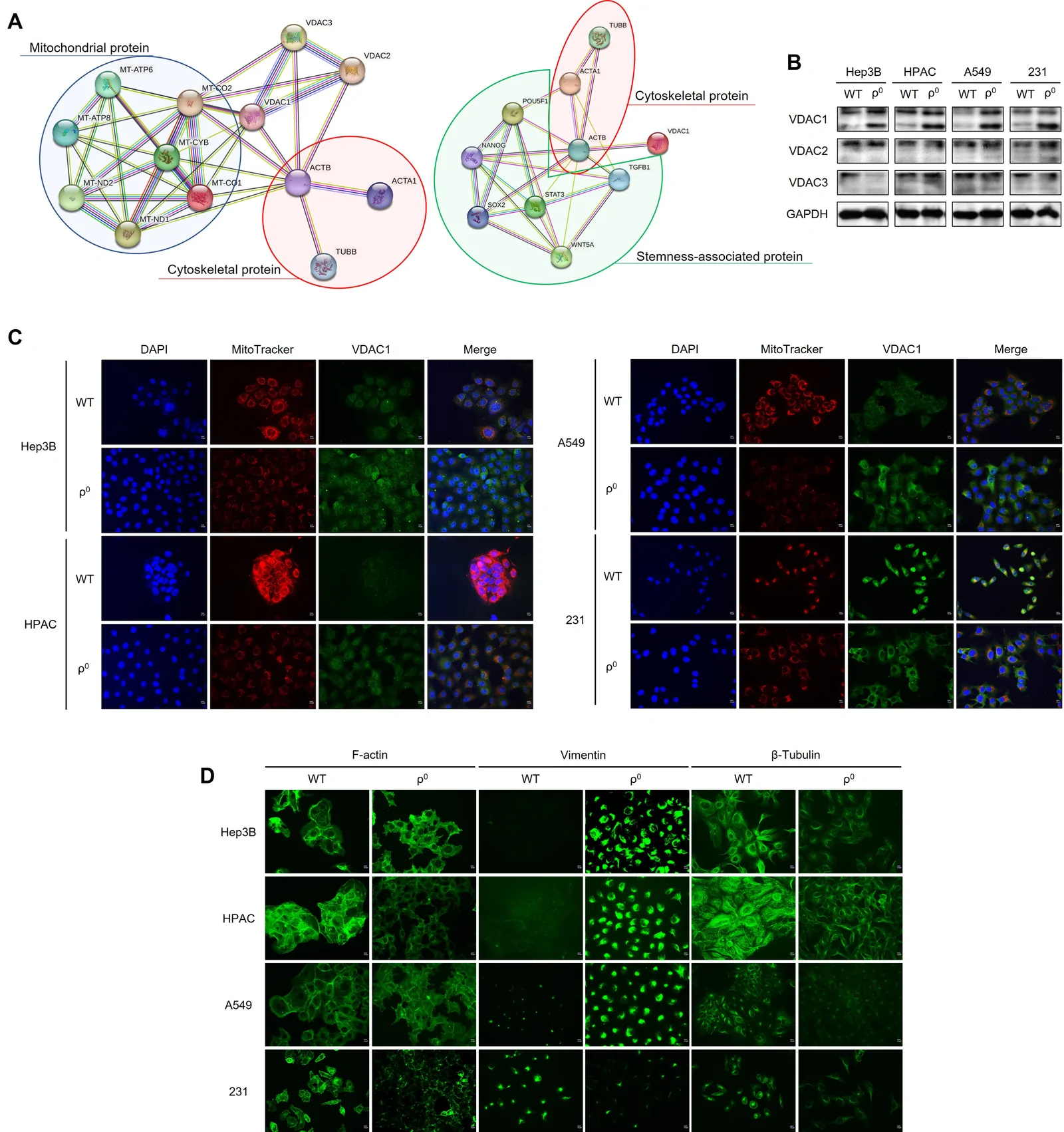
** VDAC1 expression and cytoskeletal alterations in chronically established ρ^0^ cells. (A)** STRING-based protein-protein interaction network analysis was performed using selected mitochondrial, cytoskeletal, membrane-associated, and stemness-related factors to identify candidate molecules associated with the phenotypic changes observed in ρ^0^ cells. Members of the VDAC family were identified as candidate proteins within these networks. **(B)** Expression of VDAC1, VDAC2, and VDAC3 was examined in WT and ρ^0^ cells, revealing that only VDAC1 was consistently upregulated in all ρ^0^ cell lines. **(C)** VDAC1 localization was evaluated by immunofluorescence staining using an anti-VDAC1 antibody together with MitoTracker, showing mitochondrial localization in both WT and ρ^0^ cells. **(D)** Cytoskeletal organization was assessed by immunofluorescence staining of actin, vimentin, and β-tubulin. Decreased actin and β-tubulin staining intensities were observed in all ρ^0^ cell lines, whereas vimentin staining intensity increased in most ρ^0^ cells except MDA-MB-231/ρ^0^ cells. 231 denotes MDA-MB-231.

To identify candidate molecules potentially associated with the phenotypic changes observed in ρ^0^ cells, we performed STRING-based protein-protein interaction network analysis focusing on candidate proteins related to mitochondrial dysfunction-related pathways, cytoskeletal remodeling, and stemness- and aggressiveness-associated features. Members of the VDAC family were identified as candidate proteins associated with mitochondrial proteins, cytoskeletal components, and stemness-associated proteins (Figure 3A).

We therefore examined the expression of VDAC1, VDAC2, and VDAC3 in multiple ρ^0^ cell lines by western blotting. Among the three VDAC isoforms, only VDAC1 was consistently upregulated in all ρ^0^ cell lines, whereas the expression patterns of VDAC2 and VDAC3 varied depending on the cell line (Figure 3B). These results identified VDAC1 upregulation as a common molecular feature associated with mitochondrial dysfunction and prompted us to focus on VDAC1 in subsequent analyses.

To determine the subcellular localization of VDAC1 in ρ^0^ cells, immunofluorescence staining was performed using MitoTracker and an anti-VDAC1 antibody. VDAC1 staining overlapped with mitochondrial staining, indicating mitochondrial localization in both WT and ρ^0^ cells (Figure 3C).

To determine whether the common morphological changes induced by mitochondrial dysfunction were associated with cytoskeletal remodeling, we examined representative components of the three major cytoskeletal systems: actin for microfilaments, vimentin for intermediate filaments, and β-tubulin for microtubules. Immunofluorescence analysis showed that all ρ^0^ cell lines exhibited reduced actin staining intensity and altered actin organization compared with WT cells (Figure 3D). Vimentin staining intensity was increased in most ρ^0^ cell lines, with the exception of MDA-MB-231/ρ^0^ cells, whereas β-tubulin staining intensity was consistently decreased in all ρ^0^ cell lines. These findings indicate that mitochondrial dysfunction is accompanied by cytoskeletal remodeling, characterized by reduced actin and β-tubulin staining and cell line-dependent changes in vimentin staining intensity.

Collectively, these results identify VDAC1 upregulation and cytoskeletal remodeling as common features of chronically established ρ^0^ cells.

### VBIT-4 attenuates multiple stemness-associated and aggressive phenotypes in ρ^0^ cells

**Figure 4:**
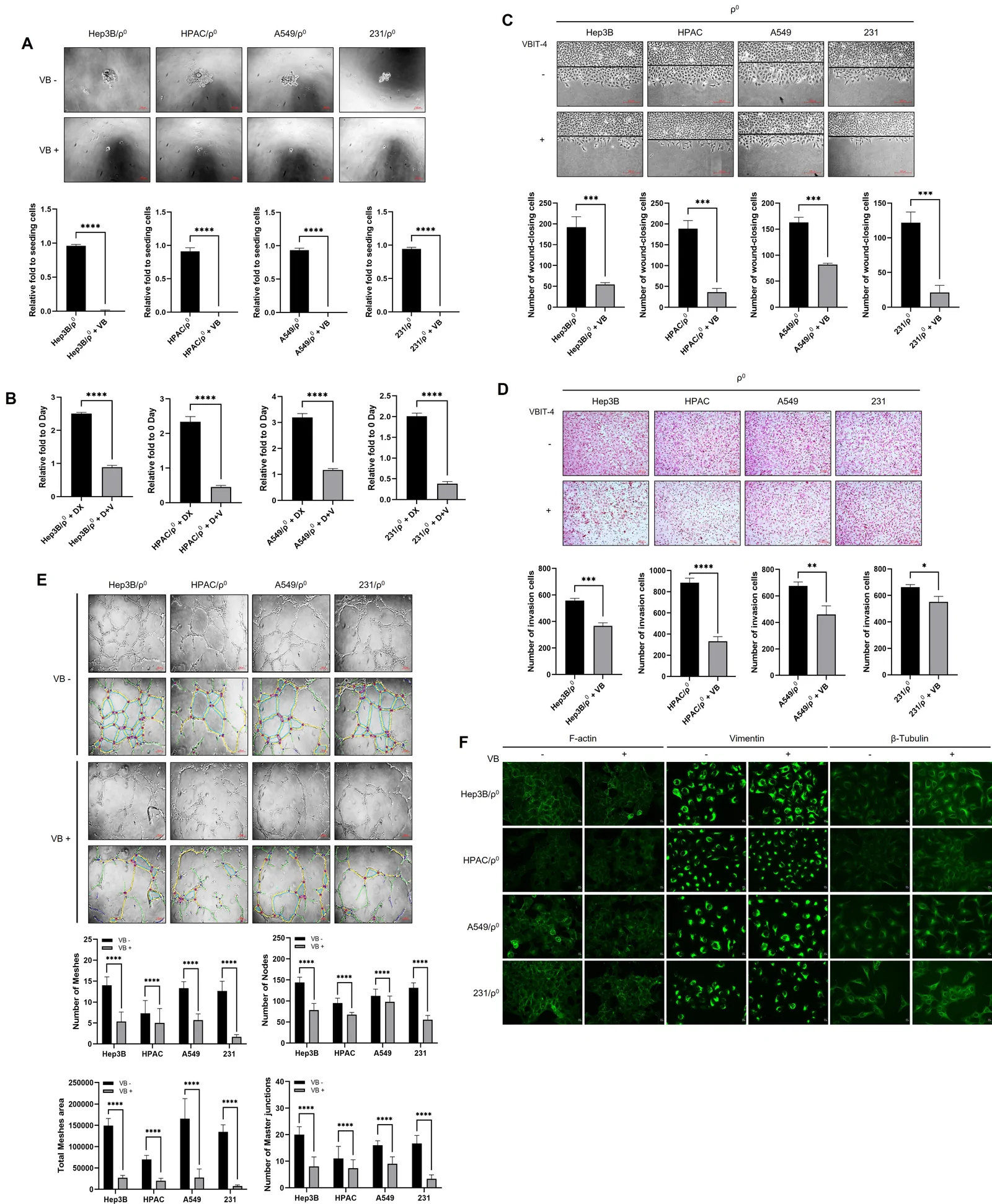
** Effects of VBIT-4 on selected stemness-associated and aggressive phenotypes in ρ^0^ cells. (A)** VBIT-4 treatment markedly reduced sphere formation in ρ^0^ cells, as shown by sphere images and quantification of sphere-forming wells. **(B)** Effects of combined VBIT-4 and DX treatment on chemoresistance varied across ρ^0^ cell lines, as assessed by crystal violet assay. **(C)** VBIT-4 significantly attenuated wound closure in ρ^0^ cells in the wound-healing assay. **(D)** VBIT-4 treatment reduced the number of invading ρ^0^ cells in the Transwell assay. **(E)** Angiogenic activity induced by ρ^0^-derived conditioned media was reduced following VBIT-4 treatment, as shown by representative tube formation images and quantitative ImageJ-based analysis. **(F)** Immunofluorescence staining showed increased β-tubulin staining intensity, a modest reduction in actin staining intensity, and no marked change in vimentin staining intensity following VBIT-4 treatment in ρ^0^ cells. VB and V represent VBIT-4; - and + indicate the absence or presence of VBIT-4 treatment, respectively. 231 denotes MDA-MB-231. Data are presented as mean ± SD. ****P < 0.0001; ***P < 0.001; **P < 0.01; *P < 0.05; ns, not significant.

To determine whether selected phenotypes observed in ρ^0^ cells are sensitive to pharmacologic inhibition with VBIT-4, we treated ρ^0^ cells with VBIT-4 and evaluated its effects on phenotypic and cytoskeletal features. VBIT-4 treatment markedly suppressed sphere formation in all ρ^0^ cell lines, as shown by sphere images and quantification of sphere-forming wells (Figure 4A). These data indicate that the sphere-forming ability of ρ^0^ cells is attenuated by VBIT-4 treatment.

We next assessed whether VBIT-4 affects chemoresistance in ρ^0^ cells. The effects of combined VBIT-4 and doxorubicin treatment varied depending on the cell line. HPAC/ρ^0^ and MDA-MB-231/ρ^0^ cells exhibited a more pronounced reduction in chemoresistance following combined treatment, whereas Hep3B/ρ^0^ and A549/ρ^0^ cells exhibited comparatively more limited responses under the same conditions (Figure 4B). These findings suggest that the effects of VBIT-4 on chemoresistance-related responses are cell line-dependent and are not uniformly observed across all ρ^0^ models.

Because wound closure and invasion were enhanced in ρ^0^ cells, we further examined whether these phenotypes were affected by VBIT-4 treatment. Wound-healing assays showed that VBIT-4 treatment significantly reduced wound closure in ρ^0^ cells (Figure 4C). Similarly, the Transwell invasion assay showed that VBIT-4 treatment reduced the number of invading ρ^0^ cells (Figure 4D). These results indicate that VBIT-4 attenuates wound closure and invasion-related phenotypes in ρ^0^ cells.

We also examined whether VBIT-4 affects the angiogenic activity induced by ρ^0^ cells. Tube formation assays showed that VBIT-4 treatment reduced angiogenic activity, as demonstrated by representative images and quantitative analysis using ImageJ (Figure 4E). The numbers of nodes, master junctions, and meshes, as well as the total mesh area, were all significantly reduced following VBIT-4 treatment, indicating that VBIT-4 attenuates the angiogenic activity induced by ρ^0^ cells.

Finally, because VDAC1 was identified together with cytoskeleton-associated proteins and ρ^0^ cells exhibited common cytoskeletal alterations, we examined whether VBIT-4 treatment affects cytoskeletal organization. Immunofluorescence analysis showed no marked change in vimentin staining, a modest decrease in actin staining, and an increase in β-tubulin staining in ρ^0^ cells following VBIT-4 treatment (Figure 4F). These findings suggest that VBIT-4 treatment is accompanied by selective changes in cytoskeletal features, particularly in β-tubulin. Collectively, these results indicate that VBIT-4 treatment attenuates several stemness-associated and aggressive phenotypes of ρ^0^ cells, although its effects on chemoresistance-related responses appear to be cell line-dependent.

## Discussion

Although mitochondrial dysfunction is recognized as a hallmark of cancer, its contribution to cancer cell plasticity remains incompletely understood 4, 5. In the present study, we established mtDNA-depleted (ρ^0^) models from pancreatic, lung, and breast cancer cell lines and found that these cells consistently exhibited common structural features together with multiple stemness-associated and aggressive phenotypes. These findings extend our previous observations in Hep3B cells and suggest that chronically established ρ^0^ cells exhibit shared stemness-associated and aggressive phenotypes across multiple cancer types 8, 18.

The first notable finding of this study was that ρ^0^ cells exhibited common structural and growth-related changes despite their distinct tissue origins. All ρ^0^ cell lines exhibited complete loss of mitochondrial gene expression and reduced mitochondrial content, confirming successful establishment of the ρ^0^ models. These observations suggest that chronically established ρ^0^ cells converge toward a common cellular architecture despite differences in tissue origin. Together with the increase in proliferation, this supports the notion that chronically established ρ^0^ cells converge on a common cellular state that is not restricted by the tissue background of the parental cancer cells.

Chronically established ρ^0^ cells also exhibited multiple stemness-associated and aggressive phenotypes. In particular, all ρ^0^ cell lines formed spheres from single cells, indicating enhanced sphere-forming ability that was unlikely to result from bulk aggregation. The Wnt-related changes were not completely uniform across the cell lines: HPAC/ρ^0^ cells exhibited increased Wnt3 expression, whereas the other ρ^0^ models generally exhibited decreased Wnt3 and increased Wnt5 expression. In addition, ρ^0^ cells exhibited reduced apoptotic responses to doxorubicin, increased ABCB1 expression, enhanced wound closure and invasion, and increased angiogenic activity. Together, these results support a broader shift in chronically established ρ^0^ cells toward a more aggressive and phenotypically plastic state 8, 18.

Among the molecular changes identified in ρ^0^ cells, VDAC1 was consistently upregulated across all tested ρ^0^ cell lines. STRING-based analysis identified associations between VDAC family proteins and mitochondrial, cytoskeletal, and stemness-associated proteins. Among the three VDAC isoforms, only VDAC1 exhibited consistent upregulation in all ρ^0^ cell lines. Because VDAC1 is involved in mitochondrial permeability, metabolite transport, and apoptotic signaling, its upregulation may be associated with the chronically established ρ^0^ state 13, 14. Notably, VDAC1 expression was increased despite reduced mitochondrial content, indicating that this change is not simply attributable to increased mitochondrial abundance.

In parallel with VDAC1 upregulation, all ρ^0^ cell lines exhibited common cytoskeletal remodeling. Actin and β-tubulin staining intensities were reduced in all ρ^0^ cell lines, whereas changes in vimentin staining were cell line-dependent. In particular, MDA-MB-231/ρ^0^ cells exhibited reduced vimentin staining, in contrast to the increased vimentin staining observed in the other ρ^0^ models, indicating that this component of the remodeling response was not uniform across all cell lines. These alterations were accompanied by convergence toward a similar cellular morphology, suggesting an association between mitochondrial dysfunction and shared structural remodeling in cancer cells. Although the present study does not establish a direct mechanistic link between mitochondrial dysfunction and cytoskeletal regulation, the coordinated changes in VDAC1, cellular morphology, and cytoskeletal organization suggest that these events are closely associated 16, 17.

Treatment with VBIT-4 attenuated multiple phenotypes observed in ρ^0^ cells. Sphere formation, wound closure, invasion, and angiogenic activity were all reduced following VBIT-4 treatment. In contrast, the effect of VBIT-4 on chemoresistance was cell line-dependent, indicating that not all phenotypes associated with the ρ^0^ state are regulated in the same manner. Together, these findings suggest that several phenotypes associated with the ρ^0^ state are sensitive to VBIT-4 treatment.

VBIT-4 treatment was also accompanied by selective cytoskeletal changes, particularly increased β-tubulin staining intensity and a modest reduction in actin staining, whereas vimentin staining was not markedly altered. These observations suggest that VBIT-4 treatment is accompanied by changes in selected structural features of ρ^0^ cells. In particular, β-tubulin staining intensity increased following VBIT-4 treatment; however, the relationship between this change and the common morphology of ρ^0^ cells remains to be clarified.

This study has several limitations. Because the experiments were performed using chronically established ρ^0^ cells, the findings should be interpreted in the context of cellular remodeling associated with long-term mtDNA depletion. In addition, although sphere-forming ability was consistently observed, additional functional assays assessing self-renewal capacity were not performed. Finally, although VBIT-4 treatment attenuated several phenotypes in ρ^0^ cells, the relationships among the ρ^0^ state, VDAC1 upregulation, and cytoskeletal remodeling remain to be further defined. Further studies, including *in vivo* validation, are needed to extend these findings.

In conclusion, chronically established ρ^0^ cancer cells from different tissue origins showed common structural changes together with multiple stemness-associated and aggressive phenotypes. These changes were accompanied by VDAC1 upregulation and cytoskeletal remodeling, and several of these phenotypes were attenuated by VBIT-4 treatment. Our findings suggest that VDAC1 upregulation is associated with the chronically established ρ^0^ state and that several phenotypes observed in ρ^0^ cells are sensitive to VBIT-4 treatment.

## Figures and Tables

**Table 1:** Primer sequences used for RT-PCR

Gene name	Sequence
COX1	5ʹ-TTG AAC AGT CTA CCC TCC C-3ʹ
	5ʹ-GCT CAC ACG ATA AAC CCT-3ʹ
COX2	5ʹ-CGT CTG AAC TAT CCT GCC-3ʹ
	5ʹ-GTC GTG TAG CGG TGA AAG-3ʹ
COX3	5ʹ-GAA AGC ACA TAC CAA GGC-3ʹ
	5ʹ-GCG GAT GAA GCA GAT AGT-3ʹ
ND1	5ʹ-GAG CAG TAG CCC AAA CAA-3ʹ
	5ʹ-TAG GGT GAG TGG TAG GAA GT-3ʹ
ND2	5ʹ-ACC CGT CAT CTA CTC TAC CA-3ʹ
	5ʹ-TAA TCC ACC TCA ACT GCC-3ʹ
CYTB	5ʹ-CAC TCC ACC TCC TAT TCT TG-3ʹ
	5ʹ-CTT ACT GGT TGT CCT CCG AT-3ʹ
ATP6	5ʹ-GTT CGC TTC ATT CAT TGC-3ʹ
	5ʹ-TGA GTA GGC TGA TGG TTT C-3ʹ
GAPDH	5ʹ-ATC TTC CAG GAG CGA GAT CCC-3ʹ
	5ʹ-AGT GAG CTT CCC GTT CAG CTC-3ʹ

## Data Availability

The data generated and/or analyzed during the current study are available from the corresponding author on reasonable request.

## Abbreviations

mtDNA: mitochondrial DNA
VDACs: voltage-dependent anion channels
DMEM: Dulbecco's modified Eagle's medium
FBS: fetal bovine serum
DX: doxorubicin
HUVEC: human umbilical vein endothelial cell
RT-PCR: reverse transcriptase-polymerase chain reaction
CM: conditioned medium
WT: wild-type
SD: standard deviation
ANOVA: analysis of variance
